# Awn Image Analysis and Phenotyping Using BarbNet

**DOI:** 10.34133/plantphenomics.0081

**Published:** 2023-08-04

**Authors:** Narendra Narisetti, Muhammad Awais, Muhammad Khan, Frieder Stolzenburg, Nils Stein, Evgeny Gladilin

**Affiliations:** ^1^ Leibniz Institute for Plant Genetics and Crop Plant Research (IPK), Molecular Genetics, 06466 Seeland, Germany.; ^2^ Leibniz Institute for Plant Genetics and Crop Plant Research (IPK), Genebank, 06466 Seeland, Germany.; ^3^ Harz University of Applied Sciences, Automation and Computer Sciences Department, 38855 Wernigerode, Germany.; ^4^ Center of integrated Breeding Research (CiBreed), Department of Crop Sciences, Georg-August-University, 37075 Göttingen, Germany.

## Abstract

Consideration of the properties of awns is important for the phenotypic description of grain crops. Awns have a number of important functions in grasses, including assimilation, mechanical protection, and seed dispersal and burial. An important feature of the awn is the presence or absence of barbs—tiny hook-like single-celled trichomes on the outer awn surface that can be visualized using microscopic imaging. There are, however, no suitable software tools for the automated analysis of these small, semi-transparent structures in a high-throughput manner. Furthermore, automated analysis of barbs using conventional methods of pattern detection and segmentation is hampered by high variability of their optical appearance including size, shape, and surface density. In this work, we present a software tool for automated detection and phenotyping of barbs in microscopic images of awns, which is based on a dedicated deep learning model (BarbNet). Our experimental results show that BarbNet is capable of detecting barb structures in different awn phenotypes with an average accuracy of 90%. Furthermore, we demonstrate that phenotypic traits derived from BarbNet-segmented images enable a quite robust categorization of 4 contrasting awn phenotypes with an accuracy of >85%. Based on the promising results of this work, we see that the proposed model has potential applications in the automation of barley awns sorting for plant developmental analysis.

## Introduction

Awns are bristle-like extensions of the glumes and/or husks of many grass species including the major crop plants such as wheat, rice, barley, and rye. The awns provide protection against pests and foraging animals. They are considered important due to their physiological role in photosynthesis [1]. Barley plants, for example, typically have long, thin awns extending from the outer husk called “lemma”.

Most awns bear barb-like structures on the surface that primarily aid in the dispersal of the plant’s seeds by facilitating, in the case of wild grass species, the adherence to the animal furs [2]. The barbs are upward-oriented single-celled trichomes with highly silicified cell walls [3] having different sizes and structures. Their presence gives awns a rough texture that can be harmful during manual harvest or lowers barley quality when producing barley as a feed crop. The barbed barley awn is called a “rough” awn. These cultivars bear large and more dense barbs covering the awns from the apical to the basal part. However, the density and size of the barbs vary across the species and cultivars, defining the intensity of awn roughness. Individual barley cultivars completely lack any barbs, except a few at the apical part of the awns, giving the awns a completely smooth surface texture and cultivars are referred to as smooth awn barley.

To look into the genetic control of the intensity of barb formation in barley, a genome-wide association study (GWAS) was conducted on 1,000 barley accessions. Two highly important loci were associated with the awn roughness trait and one major gene HvRaw1 could be isolated [4].

For the genetic mapping of the second genetic locus and to gain insight into the independent and interactive effect of both loci, we conducted a genetic mapping study using a biparental F2 population derived from a cross between a rough variety “Barke” and a smooth mutant “MHOR597”, both of which were confirmed to carry different allelic states at the 2 loci controlling awn roughness. By genotyping, we confirmed the segregation of all expected 9 possible genetic classes (own unpublished data); however, we observed only 4 different phenotypic classes of awn roughness, bearing barbs of varying density and structure, which is explained by the dominant inheritance of the wild-type alleles of both genes and the additive epistasis between both loci (own unpublished data).

To enable high-throughput screening with the unbiased classification of all different phenotypic classes, we evaluated different techniques of awn imaging to measure barb structure and density. One approach, scanning electron microscopy, allows one to visualize barbs at the highest resolution and a great level of detail [4]. These techniques allow researchers to analyze the size, shape, and arrangement of the barbs on the surface of plant awns, as well as other features such as the presence of glandular structures or the distribution of pigments.

Barbs can also be monitored using conventional light microscopy; however, here they are only distinguishable on the edges of the awns (see Fig. 2A). For analysis of visible light images, conventional analytic tools such as ImageJ [5] and GNU Image Manipulation Program (GIMP) [6] can be used. However, accurate segmentation and quantification of small, semi-transparent and often also occluding barb structures are challenging and time-consuming. In the absence of appropriate software tools, analysis of barbs in awn images (e.g., counting and measurement) is widely done manually. This approach requires a lot of time and human effort and can be hardly scaled to large datasets. Therefore, automated image processing algorithms are required to detect, quantify, and classify the barb structures in different crop plants.

Since we are interested not only in the detection and counting of barbs but also in a more comprehensive assessment of barb morphology, a consistent image segmentation approach was used in this study. The accurate segmentation of barbs like tiny objects is a critical and challenging task because of its limited spatial resolution, low contrast, and large variability. Deep learning-based methods have emerged as powerful tools for segmenting small objects like cells [7], nuclei [8], and subcellular structures [9] in biomedical images, because they can learn complex features and patterns that are difficult to capture with traditional image analysis methods. In the case of plant image analysis, several approaches were proposed for the detection of plant organs like roots [10–12], shoots [13,14], and flowers [15] and their high-throughput phenotyping. However, very few studies for automated analysis of small and optically variable plant organs, such as grain spikes [16,17], were presented in the literature. To the best of our knowledge, no appropriate tools suitable for accurate barb segmentation in awn images are known.

This paper presents a deep learning, convolutional neural network (CNN)-based approach to the segmentation of barbs in microscopic images of barley cultivars. For the task of image segmentation, a well-known encoder–decoder CNN architecture with fully convolutional layers is used. Our approach to barb segmentation is based on the extension of the U-net segmentation model from Ronneberger et al. [18]. Here, we present our methodological framework including the proposed U-net-based framework for barb detection, ground truth data generation, and training and evaluation procedures. Then, the results of experimental investigations are presented, including a model performance by application to the segmentation of test images and genotypic–phenotypic analysis.

## Materials and Methods

### Plant material

In this study, we used an F2 population that was segregating for 2 independent rough awn loci: Raw1 (A) on chromosome 5H and Raw7HS (B) on chromosome 7H, which were previously detected in a GWAS study [4]. The population was derived from the cross between rough awn variety “Barke”, wild type at both loci (AABB), and the smooth awned, x-ray mutagenized, mutant named “MHOR597” (GBIS/I, IPK Genebank) carrying the recessive mutant alleles at both loci (aabb). For image analysis, we selected 70 plants based on their either homozygous genotypic constitution at either locus. The plants were grown under controlled greenhouse conditions: 21 °C day and 17 °C night temperature, with 16 h duration of artificial light.

### Image data

**Fig. 1.**
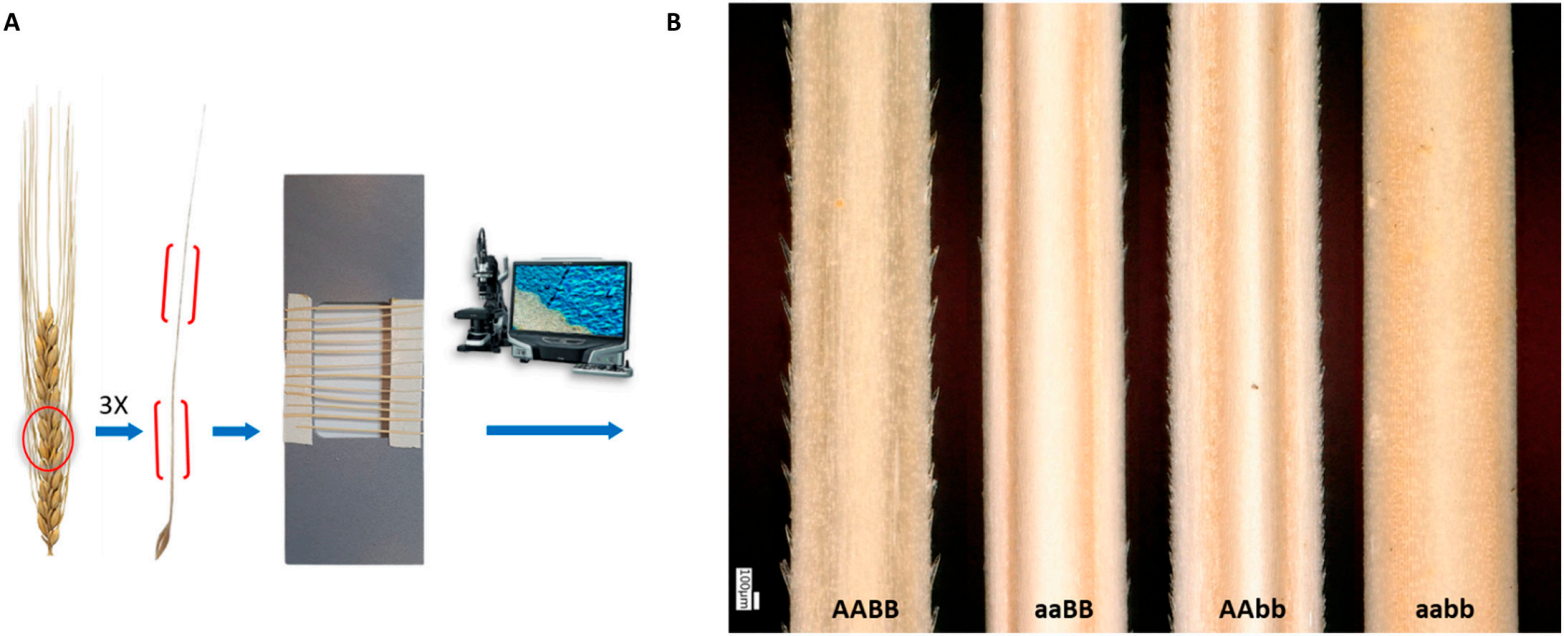
Awn imaging. (A) The central 3 awns were collected from the main spike of each plant. The central and basal parts of the awns were taped on an imaging slide and the micrographs were generated under a digital microscope. (B) The representative micrographs of the adaxial side of the basal part of the awns. All possible homozygous genetic classes for the 2 alternative alleles at the 2 awn roughness controlling loci (“A” and “B”) show varying density and size of barbs.

The roughness of the awn surface is determined by the density of the barbs, which is controlled independently by each of the 2 mentioned loci. Therefore, we needed to perform robust phenotyping by optically assessing the awns at specific “central” and “basal” locations. To achieve this, we collected 3 awns from the center of the main tiller spikes of each plant at the harvest stage and taped them on an imaging slide. We used a digital microscope (Keyence VHX) to capture images of the adaxial side of the awns at 100× magnification (Fig. 1).

### Genotyping

To study the association between barb density and the allelic state of the plants at both loci, the genotyping was performed using Kompetitive Allele-Specific PCR markers (from PACE® at 3crbio.com), which enable bi-allelic scoring at both loci.

### Ground truth generation

**Fig. 2.**
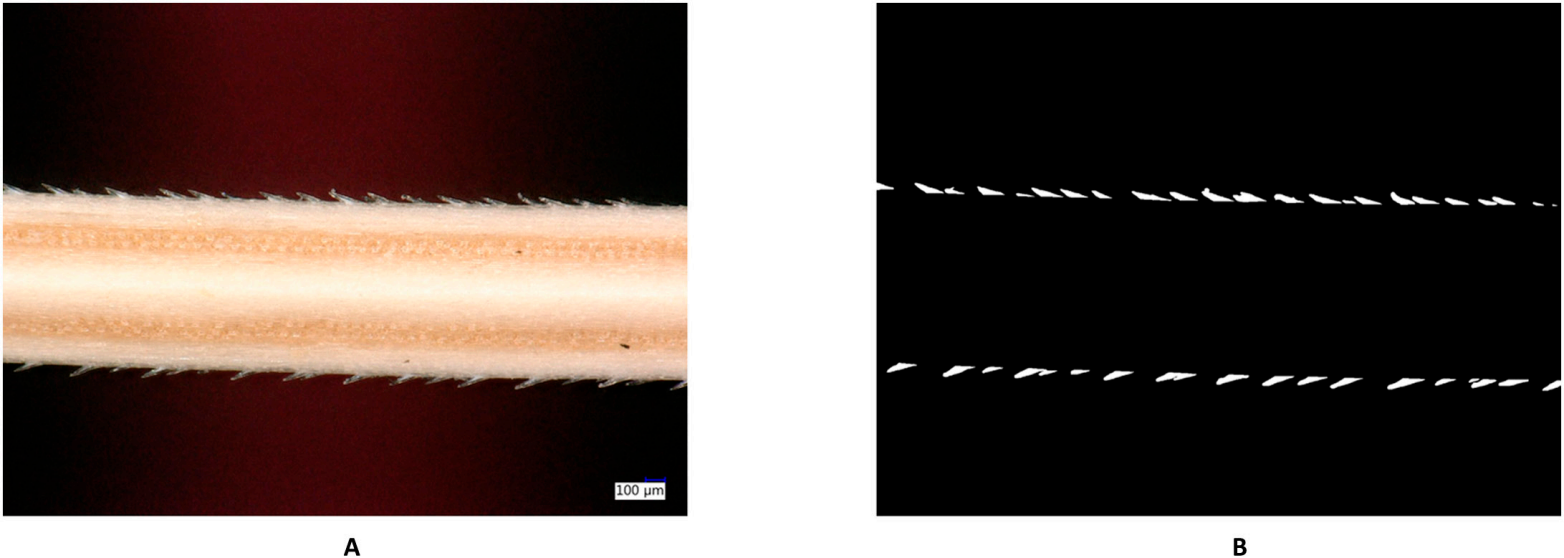
Example of barley awn image captured under the microscope. (A) Original image with the resolution of 1,200 × 1,600. (b) Ground truth mask generated by ImageJ.

In order to develop CNN segmentation models, a representative set of ground truth images with an accurate annotation of fore- and background image regions is required. In this study, ground truth images of different microscopic awn images were generated using ImageJ [5]. This tool contains an image-filling option in the toolbar that allows for the efficient annotation or filling of image regions by manually drawing a polygon around each barb in the image. Figure 2B shows an example of generated ground truth image. Manual annotation of barbs using ImageJ takes between 10 and 30 min per image depending on the number and structural complexity of the barbs of a given awn image.

### Barb detection using CNN

The BarbNet model is a modified version of the original encoder–decoder CNN architecture of U-net [18], which is designed for semantic segmentation of barley awn barbs. Unlike the U-net model, BarbNet includes batch normalization [19] after each convolution layer to enhance network performance and stability by normalizing the feature maps at respective levels [19,20]. Dropout layers are not used in BarbNet because combining batch normalization and dropout layers can lead to poor results [21]. Additionally, the kernel size is increased to improve the segmentation quality of varying and elongated target patterns [22]. Finally, the depth of BarbNet is increased to 5 compared to the original U-net depth of 4 due to the larger input image size. Table 1 provides a detailed comparison of convolutional parameters with respect to the original U-net.

**Table 1.** Convolutional parameters of the original U-net and BarbNet.

Convolutional parameters	Original U-net	BarbNet
**Kernel size**	3 × 3	5 × 5
**Transposed kernel size**	2 × 2	3 × 3
**Stride**	1 × 1	2 × 2
**Padding**	Unpadded	Padding with zeros
**Depth**	4	5
**Number of filters**	(64, 128, 256, 512, 1,028)	(16, 32, 64, 128, 256, 512)

The U-net framework was utilized for the task of barb detection on barley awns by incorporating the suggested modifications. Training and testing phases of the network were conducted on input images at their original resolution of 1,024 × 1,600.

**Fig. 3.**
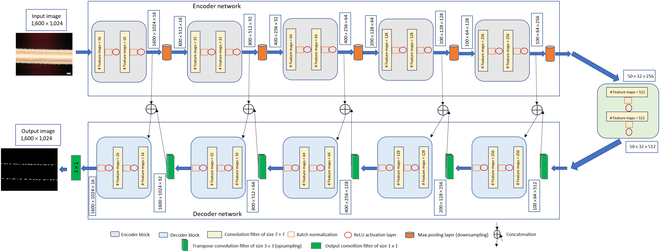
The proposed U-net architecture for barb detection on barley awn images.

The encoder network is responsible for extracting features from input image patches. It consists of 5 encoder blocks, each with 2 convolutional layers with 5 × 5 filters, followed by batch normalization [19] and rectified linear unit (ReLU) activation function [23]. Max-pooling operations are used to downsample the feature maps by half of their original size [24,25]. These steps enable a more efficient aggregation of image features. The bridge encoder block without a max-pooling layer is applied to generate 512 feature maps of size 32 × 50. The decoder network is responsible for upsampling the feature maps and generating the final output. It is composed of 4 decoder blocks, where the output from the bridge encoder is upsampled using 3 × 3 transpose convolution and stride 2. The resulting feature map is concatenated with the corresponding encoder feature maps and subsequently passed through a convolutional layer with decreasing channel depth (128, 64, 32, and 16). The output of the final decoder block is fed into a convolutional layer with the logistic function [26] to classify each pixel as barb or non-barb in the image. The output of the proposed architecture is a probability image with values ranging between 0 and 1 of size 1,024 × 1,600, similar to the input image shown in Fig. 3. Overall, the U-Net-based model demonstrates strong performance in detecting barb pixels in images.

### Performance metrics

During the training and testing phase, the performance of the proposed model (BarbNet) is evaluated using binary cross-entropy loss function [25] and the Dice coefficient (DC) [27]. The binary cross-entropy loss function compares each pixel prediction (0: non-barb, 1: barb) with the ground truth pixel and averaged all pixels losses to calculate the total image loss in the training stage.

$$\text{Binary cross}-\text{entropy loss}=-1/N\sum_{i=1}^{N}\left(Y_{i}.\text{log}{\hat{Y}}_{i}+(1-Y_{i}).\text{log}(1-{\hat{Y}}_{i})\right)$$ (1)

The DC quantifies the similarity between the model’s predicted segmentation and the ground truth segmentation, with values ranging from 0 to 1. A DC value of 0 indicates completely false segmentation (0% similarity), while a DC value of 1 indicates perfect segmentation (100% similarity). It is calculated as 2 times the area of overlap divided by the total number of pixels in both the model-predicted and ground truth binary images.

$$\mathrm{DC}=\frac{2*(\hat{Y}\cap Y)}{\hat{Y}\cup Y}=\frac{2*\sum_{i=1}^{N}‍{\hat{y}}_{i}y_{i}}{\sum_{i=1}^{N}‍{\hat{y}}_{i}+\sum_{i=1}^{N}‍y_{i}}$$ (2)

The symbols $\hat{Y}$ and *Y* represent the predicted and ground truth binary images, respectively. The values of ${\hat{y}}_{i}$ and *y_i_* correspond to the output of pixel *i* in the predicted and ground truth binary images, with possible values of either 0 or 1.

### Computational implementation

The BarbNet model was developed under Python 3.8 using TensorFlow [28] with Keras application programming interface. Furthermore, image processing operations such as reading, cropping, and training data preparation were performed using PIL, Numpy [29], and Scikit-Image [30] packages. The model was trained on a graphics processing unit (GPU) machine with a Linux operating system (Ubuntu 20.04 LTS, Intel(R) Core (TM) i7-10700K central processing unit [CPU] @ 3.80 GHz) and NVIDIA RTX 3090 with 24 GB video random-access memory (VRAM) graphic card.

As stated above, 348 images were annotated using ImageJ to train a proposed BarbNet model on barely awn images. Afterwards, the dataset was partitioned into a training set and a validation set in the ratio of 85:15, respectively, based on our experience and literature [31,32]. Each image is cropped from the original resolution (1,200 × 1,600) to 1,024 × 1,600 and normalized in the range of [0, 1] using the min–max method to ensure feature consistency in the CNN network without losing the distribution of original data. The BarbNet’s initial weights were set randomly with zero mean and an SD of 0.05, as suggested by Krizhevsky et al. [33]. An Adam optimizer [34] was applied in the model optimization process to improve the segmentation performance on training data. Since the output of the model is a binary segmentation, the binary cross-entropy loss function [25] was used to measure the error rate of the model during the training stage. The model was trained for 100 epochs with a batch size of 12 as per system constraints. The learning rate of the Adam optimizer is initialized with 0.001 and it updates the model weights during each training iteration of the model. To avoid a too-quick convergence of the model to a suboptimal solution, a dynamic learning rate scheduler was introduced to reduce the learning rate by a factor of 0.2 until 0.0001 if the validation loss is not improved in the next 5 iterations. This results in overfitting in the case of a large learning rate and getting stuck on the suboptimal solution in the case of a too-small learning rate can be eliminated [35]. Finally, an early stopping criterion is introduced if training cross-entropy loss is not improved in the next 10 iterations.

In the next step, the optimized BarbNet model was used for the segmentation of barb structures and subsequent assessment of their phenotypic traits. The MATLAB 2021a routines performing this analysis were compiled to a single license-free executable tool that can be downloaded from electronic Data Archive Library (e!DAL).

The output layer of the model consisted of a logistic activation function, which produced a probability map ranging from 0 to 1 for the segmentation. To convert this probability map into a binary image, a threshold value, denoted as *tsh*, was chosen. Since the probability of barb pixels is higher than that of background pixels, a threshold value of *tsh* ≥ 0.5 was selected to classify all high-probability pixels as barb pixels in the final segmentation. In the post-processing step, segmented objects with an area less than 15 pixels were removed to eliminate false (awn barbs) positives by the phenotypic traits calculation.

## Results

### Training and validation of BarbNet

**Fig. 4.**
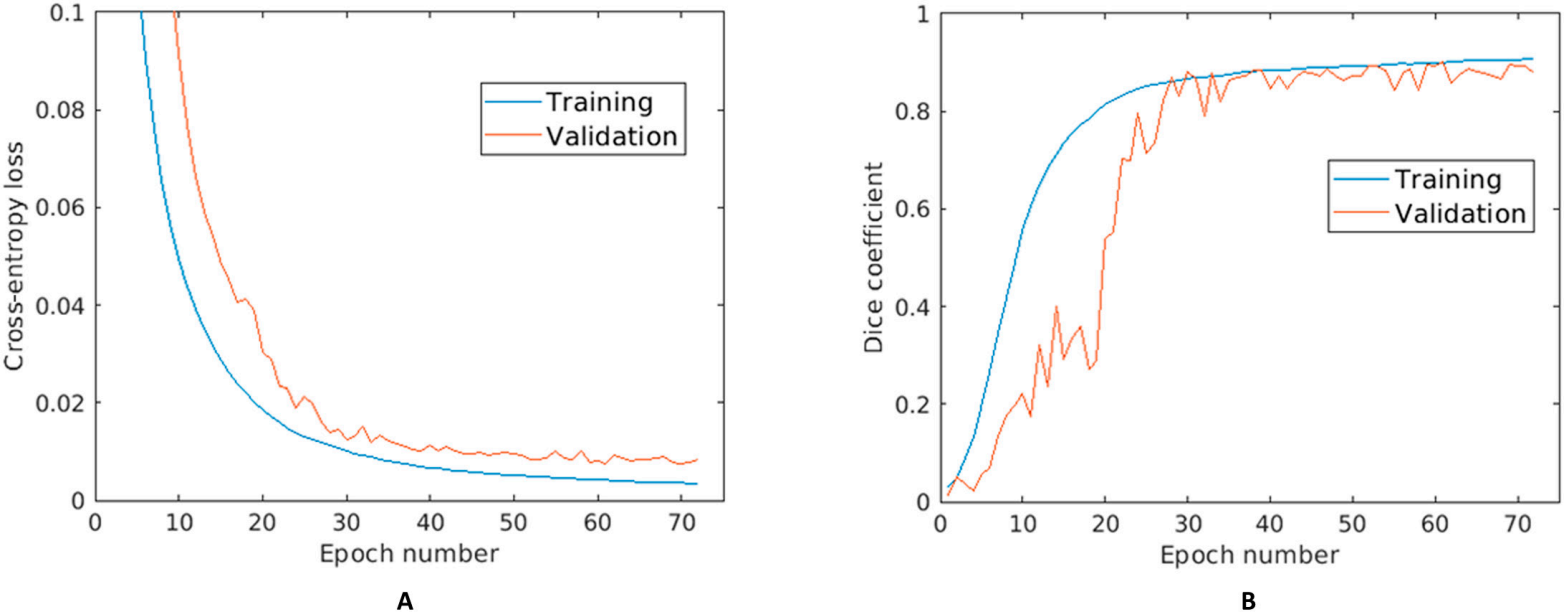
Training and validation performance of the BarbNet model over 75 epochs with respect to (A) the cross-entropy loss and (B) the Dice coefficient. *x*- and *y*-axes represent the epoch number and performance measure, respectively.

The training and validation of BarbNet were performed on a set of total 348 images that were subdivided into training and validation subsets with a ratio of 85:15, respectively. The dataset contains different awn phenotypes including barbs of different sizes and densities (smooth, sparse, moderate, and dense). During the training stage of the network, the performance of the model was analyzed using binary cross-entropy loss and DC at each epoch. Figure 4 shows the training and validation of BarbNet over 75 epochs. It shows that training loss (Fig. 4A) was minimized and flatten the curve 50 epochs. Simultaneously, training DC (Fig. 4B) achieved more than 0.91 from epoch number 50. However, the generalized performance of the optimized model is analyzed using validation metrics. The BarbNet model achieved a maximum validation DC of 0.91 and a minimum validation loss of 0.0076 at epoch number 61. Thenceforth, model training was not improved and terminated at epoch number 72.

### Evaluation and comparison of U-net models

**Fig. 5.**
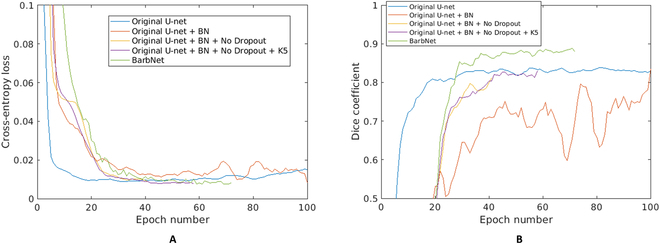
Comparison of performance metric of BarbNet vs. U-net with respect to (A) the cross-entropy loss and (B) the Dice coefficient. *x*- and *y*-axes represent the epoch number and performance measure, respectively.

The BarbNet model represents an extension of the U-net framework from Ref. [18]. In order to adapt the U-net to the task of barb (small object) segmentation, several modifications were introduced including (a) inclusion of batch normalization (original U-net + BN); (b) exclusion of dropout layers (original U-net + BN + No Dropout); (c) increased kernel size of 5 (original U-net + BN + No Dropout + K5); and (d) increased depth of the model to 5 and reduced number of filters (original U-net + BN + No Dropout + K5 + D5). The last U-net modification (d) turned out to show that the best performance of barb segmentation is further termed as BarbNet. For the evaluation and comparison of these models, the same image sets for training and validation were used. Figure 5 shows a comparison of performance of all 4 modified U-net models vs. the original U-net for the task of barb segmentation. It shows that the validation loss of both “original U-net + BN + No Dropout + K5” and BarbNet decreases after epoch number 40 and converges earlier than the remaining 3 U-net models with a higher validation loss. However, BarbNet outperforms all the other U-net models with respect to the Dice accuracy measure of image segmentation beginning from the epoch number 30. The details on validation metrics for all 4 modified and original U-net models are summarized in Table 2.

**Table 2.** Comparison of performance of 4 modified vs. original U-net model over 100 training epochs with respect to the cross-entropy loss and the Dice coefficient.

Model	Cross-entropy loss	Dice coefficient
**Original U-net**	0.0081	0.85
**Original U-net + BN**	0.0082	0.84
**Original U-net + BN + No Dropout**	0.0086	0.82
**Original U-net + BN + No Dropout + K5**	0.0070	0.85
**BarbNet**	0.0076	0.91

The performance of all CNN segmentation models is also evaluated on a test set of completely unseen 19 images that were not used for model training (see Table 3). It shows that BarbNet outperforms the original as well as all other modified U-net models with a mean accuracy of more than 90% for all test images. Examples of BarbNet segmented images including smooth (image number 2), sparse (image number 9), moderate (image number 11), and dense (image number 14) barb distributions can be found in the Supplementary Materials (Figs. S1, S2, S3, and S4, respectively). Thereby, the largest differences in performance between BarbNet and other U-net models were observed for the smooth awn phenotype. The average computational time required for the BarbNet to segment a 1,600 × 1,024 test image on a PC with Intel(R) Xeon(R) CPU E5-2640 2.40 GHz CPU is 1.10 s.

**Table 3.** Comparison of mean Dice coefficient of U-net models on a test dataset of 19 images.

Model	Mean Dice coefficient
**Original U-net**	0.82
**Original U-net + BN**	0.86
**Original U-net + BN + No Dropout**	0.80
**Original U-net + BN + No Dropout + K5**	0.83
**BarbNet**	0.93

### Evaluation of phenotypic traits

**Fig. 6.**
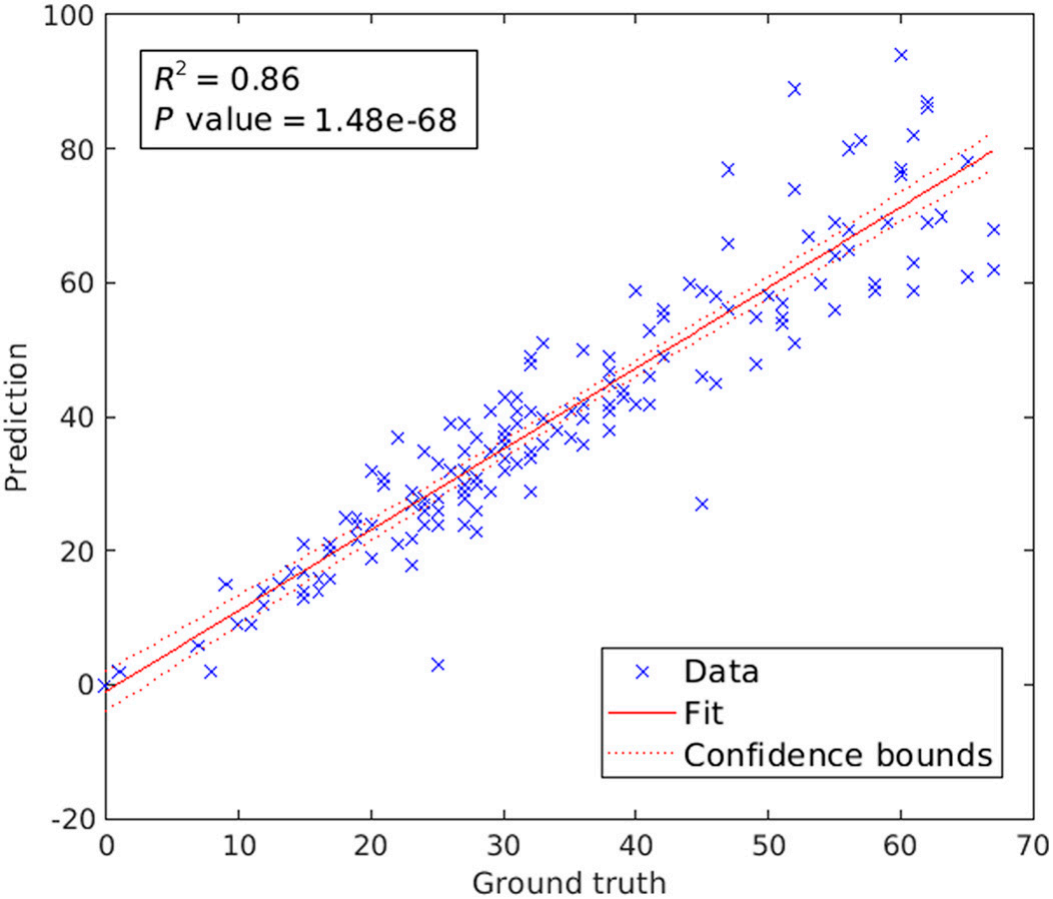
Correlation between the ground truth (*x*-axis) and predicted (*y*-axis) barb count over 156 awn images. Each point represents the total number of barbs per awn image. The red solid line and dotted lines represent a fitted curve and 95% confidence bounds, respectively. The *R*^2^ value indicates good conformity between ground truth and BarbNet count of image segmentation and trait calculation.

In addition to segmentation performance, phenotypic properties of awns calculated using our automated segmentation models were compared to manually segmented (ground truth) data. For this purpose, 10 phenotypic features are proposed in this study. Further information on the definition of traits is included in the Supplementary Materials (see Table S1). Out of 10 traits, only 3 important features for awn and barb characterization named total barb count, mean barb area, and mean barb length in pixels are presented. Figure 6 shows the correlation between the ground truth (*x*-axis) and predicted barb count (*y*-axis) over 156 awn images. Differently from the above-mentioned set of ground truth segmented 348 images, this dataset is independently prepared by biologists for evaluation of barb count predictions. Here, the *P* value represents that the BarbNet is a highly significant (<0.05) model that exhibits a higher correlation coefficient of determination (*R*^2^) value of 0.86. It indicates that BarbNet exhibits 86% conformity between the ground truth and predicted barb count.

### Genotypic–phenotypic classification

Four major awn phenotypes were observed in this study including smooth (aabb), sparse (aaBB), moderate (AABB), and dense (AAbb) on the basis of the density and size of the barbs. Here, A and B are 2 independently segregating (unlinked) genes each represented by a dominant wild-type (capital letter) and a recessive mutant (small letter) allele. In an F2 progeny, the genes may segregate among others into 4 distinct, fully homozygous genotypic classes, i.e., AABB, aaBB, AAbb, or aabb. In total, 326 awn images of basal (176) and central (150) regions from these respective 4 genotypes were used. Further details on data distribution among the 4 genotypic classes are listed in Tables 4 and 5.

**Table 4.** F1-score of 4 genotypes with count and area features.

Count vs. area	Basal (no. of images)	Central (no. of images)
**aabb**	0.89 (36)	0.91 (35)
**aaBB**	0.68 (62)	0.85 (60)
**AABB**	0.90 (40)	0.81 (32)
**AAbb**	0.75 (38)	0.85 (23)
**Mean F1-score**	0.81 (176)	0.86 (150)

**Table 5.** F1-score of 4 genotypes with count and length features.

Count vs. length	Basal (no. of images)	Central (no. of images)
**aabb**	0.96 (36)	0.97 (35)
**aaBB**	0.72 (62)	0.90 (60)
**AABB**	0.87 (40)	0.83 (32)
**AAbb**	0.69 (38)	0.82 (23)
**Mean F1-score**	0.81 (176)	0.88 (150)

**Fig. 7.**
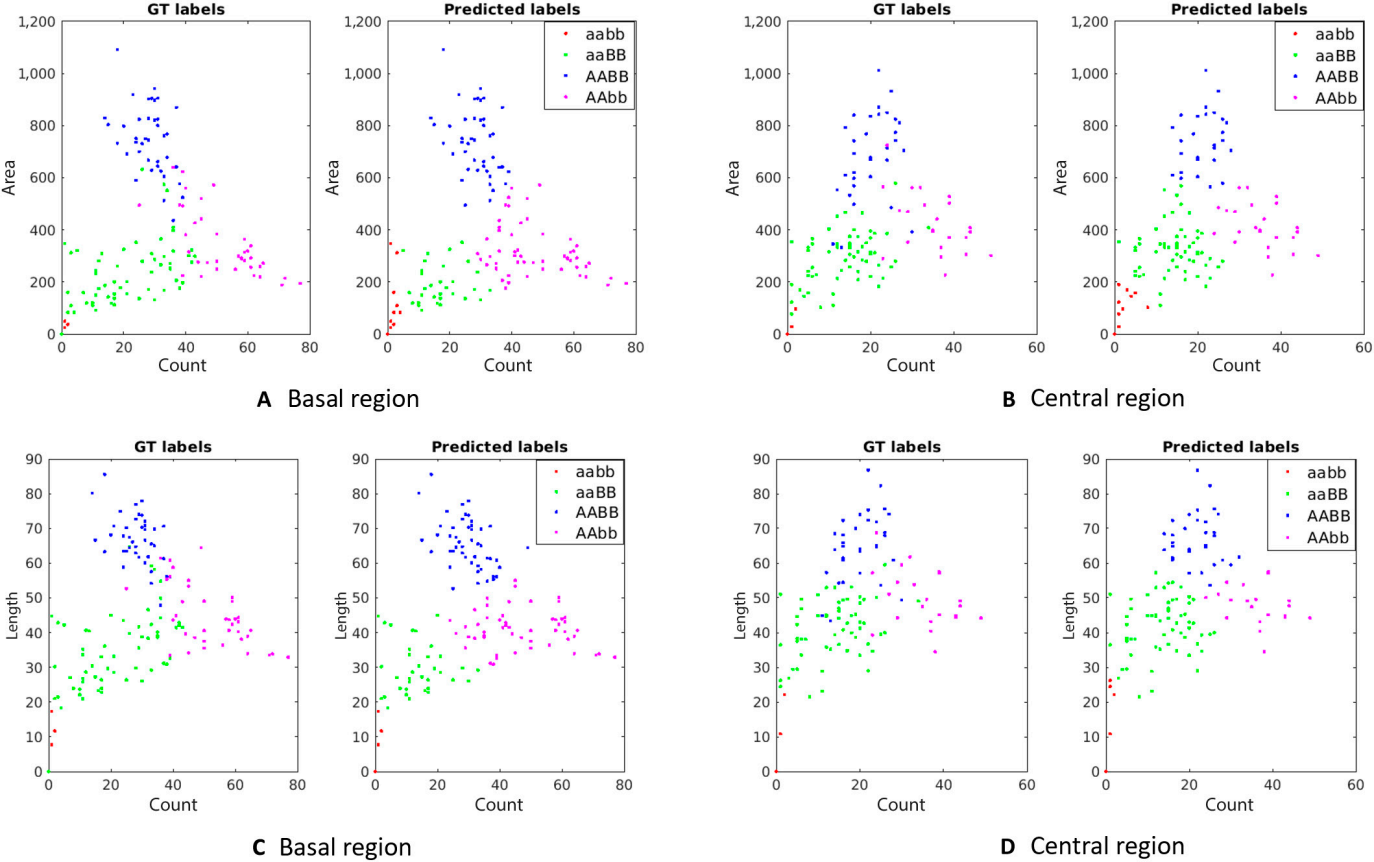
Clustering of phenotypic traits on 4 different barb distributions (smooth, sparse, moderate, and dense) using k-means. (A) Basal region: Count vs. Area, mean F1 score: 0.81. (b) Central region: Count vs. Area, mean F1 score: 0.86. (C) Basal region: Count vs. Length, mean F1 score: 0.81. (D) Central region: Count vs. Length, mean F1 score: 0.88. These 4 genotypes were well clustered with an F1-score of >85% on central regions of awns in both phenotypic traits. Area and length features are measured in pixels.

Next, we tested how accurately awn regions (basal or central) can be assigned to one of these 4 genetic classes using features derived from BarbNet-segmented images. For this purpose, 10 phenotypic features and their pairwise combinations were compared. The classification was performed using unsupervised k-means clustering after normalizing the trait values in between 0 and 1 using the min–max method, and subsequently starting with estimates of cluster centroids obtained from original density clusters as shown in Fig. 7. Our proposed prediction model accurately clustered the phenotype, confirming the precision of our predictions in classifying the phenotype into the expected number of clusters defined by the genotype. The best-performing pair of features turn out to be total barb count and barb area (average accuracy of 86%) and total barb count and barb length (average accuracy of 88%) on awns central regions (see Tables 4 and 5).

## Discussion

Awns are important for the phenotypic description of grain crops. However, accurate detection of barbs on awn surfaces is challenging because of their limited spatial resolution, low contrast, and large variability. Taking advantage of the latest advancements in deep learning, the U-net model was considered and applied to perform automatic analysis of awns in a high-throughput manner. However, such direct transfer led to unsatisfactory performance because the original U-net model was not designed for the awn image analysis. Hence, we modified the U-net architecture to fully automate the segmentation of barbs in microscopic images. Our experimental results have shown a remarkable accuracy of the modified U-net model (BarbNet) for fully automated segmentation of barbs of different sizes, shapes, and densities.

The training and validation performance of the BarbNet model has improved over the number of epochs. Besides, the model before epoch number 30 under-performed and showed the worst performance in the validation stage. However, due to the dynamic reduction in the learning rate by a factor of 0.2, a stable performance of approximately 90% DC is achieved from epoch number 50. Finally, the optimized model with a maximum validation DC of 0.91 and a minimum cross-entropy loss of 0.0076 at epoch number 61 is saved for the automated awn phenotyping.

Comparison of the original and 4 modified U-net models led to a selection of the most accurate and robust (BarbNet) crossover in all awn phenotypes. Thereby, it was observed that the original U-net as well as the “original U-net + BN” models failed to converge within the first 100 epochs. We draw the reduced accuracy of these 2 models to the presence of dropout layers removing small or fine-grained structures with low probability values, which effectively reduces the capacity of the model. This results in low-validation DC values, especially for the “original U-net + BN” model. On the other side, 3 other models with only batch normalization but without dropout layers turn out to exhibit a rapid convergence. Because batch normalization normalizes the activations, gradients of the training model are stabilized, which reduces the likelihood of vanishing or exploding gradients. As a result, a more stable gradient flow enables smoother and more efficient optimization, leading to faster convergence of these models.

In addition, by reducing the number of filters with increased depth of the model, BarbNet focuses on extracting more localized and detailed features from the overall large awn images. It results in the DC of BarbNet being improved by 6% compared to the other U-net models (Table 2). This is also reflected in the higher DC of the BarbNet model (≥90) on all unseen awn images with different barb distributions compared to other U-net models. Consequently, all 4 modified U-net models but, in particular, BarbNet exhibit a superior accuracy and robustness by segmentation of barb crossover in different awn phenotypes.

Furthermore, phenotypic characterization and classification of awns show a highly significant correlation for the barb’s count, indicating that barb detection and phenotyping using BarbNet are practically very near to the human-supervised one. However, the difference in count estimation between ground truth and BarbNet might result from occluding neighbor barbs. This may lead to a reduction in barb count per image compared to the ground truth.

Since accurate pixel-wise segmentation and not just a region detection model was used in our approach, the genotype of segmented barbs can be accurately and comprehensively characterized in terms of different phenotypic features. Our results on genotypic–phenotypic classification indicate distinct clustering of all 4 observed phenotypic classes and show its correspondence with the associated genotypic class. Moreover, it indicates that both genes contribute independently to the awn roughness trait: gene “A” controls the barb density, while gene “B” regulates the barb size. Furthermore, the location of the pink and blue clusters on each graph suggests that gene “A” plays a more important role in defining the awn roughness trait.

Similarly, our experiments with different genotypes explain that a combination of barb density and size provides the best results on central regions of awns (i.e., >85%) than basal regions (81%). In particular, the classification accuracy of genotype aaBB is improved by more than 15%. Because the basal regions of this genotype are smoother with almost no barbs than the central region, this results in less classification accuracy by unsupervised k-means algorithm. However, the accuracy reduction of 5% in the central regions of genotype AABB is due to the false positives that occurred between the moderate and dense phenotypes. Since awns have tightly connected neighborhood barbs in the dense phenotype, they tend to have higher lengths similar to the moderate barb density. Therefore, one can achieve the best and most balanced results when features of barb (area and length) and features of awns (count) are combined to perform phenotypic–genotypic analysis.

In conclusion, our approach provides an efficient solution with 90% accuracy for the detection of barbs in barley awn images. Our model turns out to be robust enough for the detection of barbs in different awn phenotypes. Even though our model exhibits quite accurate detection rates, the topic of barb and, more generally, small organ detection is still an emergent topic in the broader field of plant phenotyping. Presently, our model occasionally ignores tiny barbs, which can be attributed to the large-scale downsampling and network depth. In addition, neighbor barbs in the dense phenotypes are occluded together, leading to larger error rates in phenotypic traits. These limitations may be overcome by extending the training set of ground truth images, especially in dense and sparse phenotypes. Furthermore, alternative segmentation and region detection CNNs such as DeepLab and You Only Look Once (YOLO) can be taken into consideration for capturing small objects such as barbs and other plant organs.

## Data Availability

The datasets analyzed and source code during the current study are available from the corresponding author upon reasonable request.

## Supplementary Materials

Supplementary 1 The list of awn phenotyping features is listed in the supplementary material (Table S1) provided with the manuscript.

## References

[1] Abebe T, Wise RP, Skadsen RW. Comparative transcriptional profiling established the awn as the major photosynthetic organ of the barley spike while the lemma and the Palea primarily protect the seed. Plant Genome. 2009;2(3):0019.

[2] Elbaum R, Zaltzman L, Burgert I, Fratzl P. The role of wheat awns in the seed dispersal unit. Science. 2007;316(5823):884–886. 17495170 10.1126/science.1140097

[3] Kondorosi E, Roudier F, Gendreau E. Plant cell-size control: Growing by ploidy? Curr Opin Plant Biol. 2000;3(6):488–492. 11074380 10.1016/s1369-5266(00)00118-7

[4] Milner SG, Jost M, Taketa S, Mazón ER, Himmelbach A, Oppermann M, Weise S, Knüpffer H, Basterrechea M, König P, et al. Genebank genomics highlights the diversity of a global barley collection. Nat Genet. 2019;51(2):319–326. 30420647 10.1038/s41588-018-0266-x

[5] Huang C, Becker MF, Keto JW, Kovar D. Annealing of nanostructured silver films produced by supersonic deposition of nanoparticles. J Appl Phys. 2007;102(5):054308.

[6] The GIMP Development Team. *Gimp*, version 2.10.12, 12 Jun 2019. https://www.gimp.org

[7] Al-Kofahi Y, Zaltsman A, Graves R, Marshall W, Rusu M. A deep learning-based algorithm for 2-d cell segmentation in microscopy images. BMC bioinformatics. 2018;1:365. 10.1186/s12859-018-2375-z PMC6171227 30285608

[8] Narotamo H, Sanches JM, Silveira M. Segmentation of cell nuclei in fluorescence microscopy images using deep learning. Paper presented at: Pattern Recognition and Image Analysis: 9th Iberian Conference, IbPRIA 2019; 2019 Jul 1–4; Madrid, Spain.

[9] Sekh AA, Opstad IS, Godtliebsen G, Birgisdottir ÅB, Ahluwalia BS, Agarwal K, Prasad DK. Physics-based machine learning for subcellular segmentation in living cells. Nat Mach Intell. 2021;3(10):1071.

[10] Wang T, Rostamza M, Song Z, Wang L, McNickle G, Iyer-Pascuzzi AS, Qiu Z, Jin J. Segroot: A high throughput segmentation method for root image analysis. Comput Electron Agric. 2019;162:845–854.

[11] Narisetti N, Henke M, Seiler C, Shi R, Junker A, Altmann T, Gladilin E. Semi-automated root image analysis (saRIA). Sci Rep. 2019;9(1):19674. 31873104 10.1038/s41598-019-55876-3 PMC6928233

[12] Narisetti N, Henke M, Seiler C, Junker A, Ostermann J, Altmann T, Gladilin E. Fully-automated root image analysis (faRIA). Sci Rep. 2021;11(1):16047. 34362967 10.1038/s41598-021-95480-y PMC8346561

[13] Rakhmatulin I, Kamilaris A, Andreasen C. Deep neural networks to detect weeds from crops in agricultural environments in real-time: A review. Remote Sens. 2021;13(21):4486.

[14] Narisetti N, Henke M, Neumann K, Stolzenburg F, Altmann T, Gladilin E. Deep learning based greenhouse image segmentation and shoot phenotyping (deepshoot). Front Plant Sci. 2022;13:906410. 35909752 10.3389/fpls.2022.906410 PMC9328757

[15] Hu K. Deep learning techniques for in-crop weed identification: A review. ArXiv 2021. preprint arXiv:2103.14872.

[16] Ullah S, Henke M, Narisetti N, Panzarová K, Trtílek M, Hejatko J, Gladilin E. Towards automated analysis of grain spikes in greenhouse images using neural network approaches: A comparative investigation of six methods. Sensors. 2021;21(22):7441. 34833515 10.3390/s21227441 PMC8621358

[17] Misra T, Arora A, Marwaha S, Chinnusamy V, Rao AR, Jain R, Sahoo RN, Ray M, Kumar S, Raju D, et al. Spikesegnet—A deep learning approach utilizing encoder-decoder network with hourglass for spike segmentation and counting in wheat plant from visual imaging. Plant Methods. 2020;16:40. 32206080 10.1186/s13007-020-00582-9 PMC7079463

[18] Ronneberger O, Fischer P, Brox T. U-net: Convolutional networks for biomedical image segmentation. In: *International Conference on Medical image computing and computer-assisted intervention*. Munich (Germany): Springer; 2015. p. 234–241.

[19] Ioffe S, Szegedy C. Batch normalization: Accelerating deep network training by reducing internal covariate shift. ArXiv. 2015. 10.48550/arXiv.1502.03167

[20] Santurkar S, Tsipras D, Ilyas A, Madry A. How does batch normalization help optimization. ArXiv. 2019. 10.48550/arXiv.1805.11604

[21] Li X, Chen S, Hu X. J. Yang. Understanding the disharmony between dropout and batch normalization by variance shift. ArXiv. 2018. 10.48550/arXiv.1801.05134

[22] Peng C, Zhang X, Yu G, Luo G, Sun J. Large kernel matters—Improve semantic segmentation by global convolutional network. Paper presented at: Proceedings of the IEEE Conference on Computer Vision and Pattern Recognition (CVPR); 2017 Jul 21–26; Hawaii.

[23] Agostinelli F, Hoffman M, Sadowski P, Baldi P. Learning activation functions to improve deep neural networks. ArXiv. 2014. 10.48550/arXiv.1412.6830.

[24] Wang L, Guo S, Huang W, Qiao Y. Places205-vggnet models for scene recognition. ArXiv. 2015. 10.48550/arXiv.1508.01667

[25] Jha RR, Jaswal G, Gupta D, Saini S, Nigam A. Pixisegnet: Pixel-level iris segmentation network using convolutional encoder-decoder with stacked hourglass bottleneck. IET Biometrics. 2019;9(1):11–24.

[26] Dunne RA, Campbell NA. On the pairing of the softmax activation and cross-entropy penalty functions and the derivation of the softmax activation function. Paper presented at: Proc. 8th Aust. Conf. on the Neural Networks, Melbourne, Australia: Citeseer; 1997.

[27] Zou KH, Warfield SK, Bharatha A, Tempany CMC, Kaus MR, Haker SJ, Wells WM III, Jolesz FA, Kikinis R. Statistical validation of image segmentation quality based on a spatial overlap index1: Scientific reports. Acad Radiol. 2004;11(2):178–189. 14974593 10.1016/S1076-6332(03)00671-8 PMC1415224

[28] Abadi M, Agarwal A, Barham P, Brevdo E, Chen Z, Citro C, Corrado GS, Davis A, Dean J, Devin M, et al. Tensorflow: Large-scale machine learning on heterogeneous distributed systems. ArXiv. 2016. 10.48550/arXiv.1603.04467

[29] van de Warlt S, Colbert SC, Varoquaux G. The numpy array: A structure for efficient numerical computation. Comput Sci Eng. 2011;13(2):22–30.

[30] Van der Walt S, Schonberger JL, Nunez-Iglesias J, Boulogne F, Warner JD, Yager N, Gouillart E, Yu T. Scikit-image: Image processing in python. PeerJ. 2014;2:e453. 25024921 10.7717/peerj.453 PMC4081273

[31] Crimi A, Bakas S, Kuijf H, Menze B, Reyes M. *Brainlesion: Glioma, Multiple Sclerosis, Stroke and Traumatic Brain Injuries: Third International Workshop*, *BrainLes 2017, Held in Conjunction with MICCAI 2017, Quebec City, QC, Canada, September 14, 2017, Revised Selected Papers*; Springer; 2018.

[32] Joseph VR. Optimal ratio for data splitting. *Statistical Analysis and Data Mining: The ASA Data Science Journal*. 2022.

[33] Krizhevsky A, Sutskever I, Hinton GE. Imagenet classification with deep convolutional neural networks. In: *Advances in neural information processing systems*. Cambridge (MA): Massachusetts Institute of Technology Press; 2012. p. 1097–1105.

[34] Kingma DP, Ba J. Adam: A method for stochastic optimization. ArXiv 2014. 10.48550/arXiv.1412.6980

[35] Bengio Y. Practical recommendations for gradient-based training of deep architectures. In: *Neural networks: Tricks of the trade*. Heidelberg (Germany): Springer; 2012. p. 437–478.

