# Supplementary material for: Awn Image Analysis and Phenotyping Using BarbNet
Source: Plant Phenomics. 2023 Aug 4;5:0081. doi: 10.34133/plantphenomics.0081 (PMC10794052; doi:10.34133/plantphenomics.0081)
Supplement: Supplementary 1 — The list of awn phenotyping features is listed in the supplementary material (Table S1) provided with the manuscript. [file plantphenomics.0081.f1.pdf]

Supplementary Material

Awn image analysis and phenotyping using BarbNet

Narendra Narisetti, Muhammad Awais, Muhammad Khan , Frieder Stolzenburg, Nils Stein, and Evgeny Gladilin

Table S1. Description barb traits.

**Trait Name**	**Description**
Total Count	Total number detected barbs per awn image.
Lower Count	Number of detected barbs in the lower edge of the awn.
Upper Count	Number of detected barbs in the upper edge of the awn.
Area	Number of pixels of segmented barbs. It measures the size of barbs.
Length	Major axis length of barbs approximated by fitting ellipse to the barb object. It measures the elongated length of each barb.
Circularity	Measures the roundness of segmented barbs. It indicates the shape of barbs like circle or elliptical.
Perimeter	Measures the length of barbs boundary. It indicates how large is the size of barbs.
Compactness	Ratio of area of barb to the area of ellipse. It measures how irregular are the boundaries of barbs.
Lower_dist	Euclidian distance between the neighborhood barbs in the lower edge of the awn. It measures how close barbs are places on the awn.
Upper_dist	Euclidian distance between the neighborhood barbs in the upper edge of the awn. It measures how close barbs are places on the awn.

Figure S1. Examples of barb segmentation on smooth awn phenotype using original U-net and BarbNet: (a) original image, (b) segmentation using the original U-net vs. ground truth (DC=0), (c) segmentation using BarbNet vs ground truth (DC = 0.83). For the visualization purpose, original images were cropped to the region of interest.

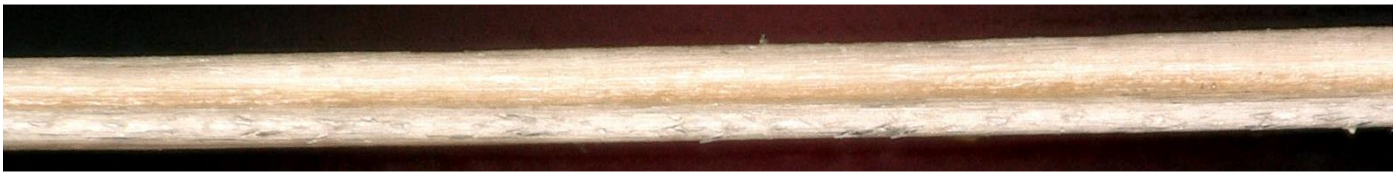

(a)

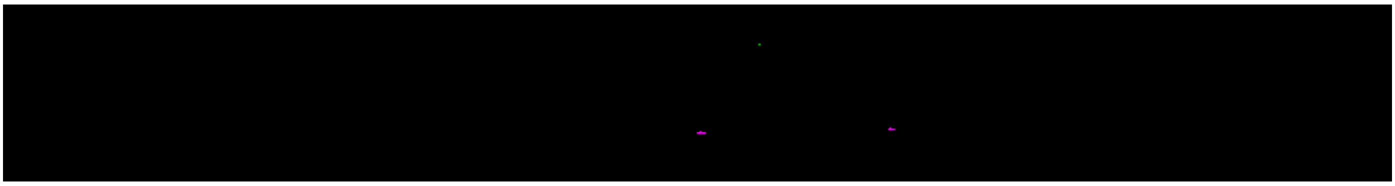

(b)

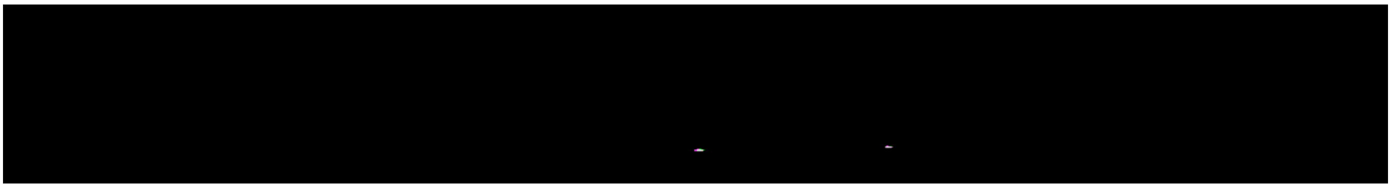

(c)

Figure S2. Examples of barb segmentation on sparse awn phenotype using original U-net and BarbNet: (a) original image, (b) segmentation using the original U-net vs. ground truth (DC=0.83), (c) segmentation using BarbNet vs ground truth (DC = 0.94). For the visualization purpose, original images were cropped to the region of interest.

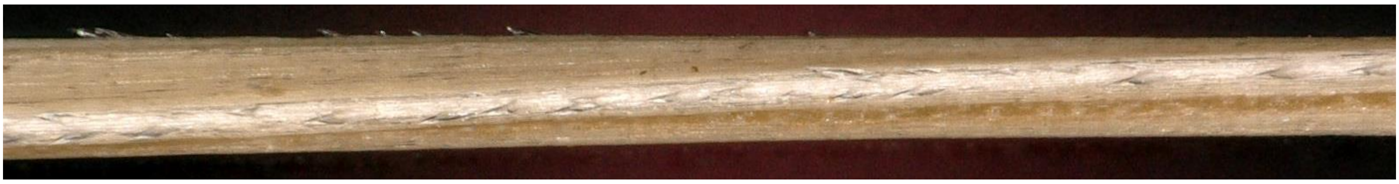

(a)

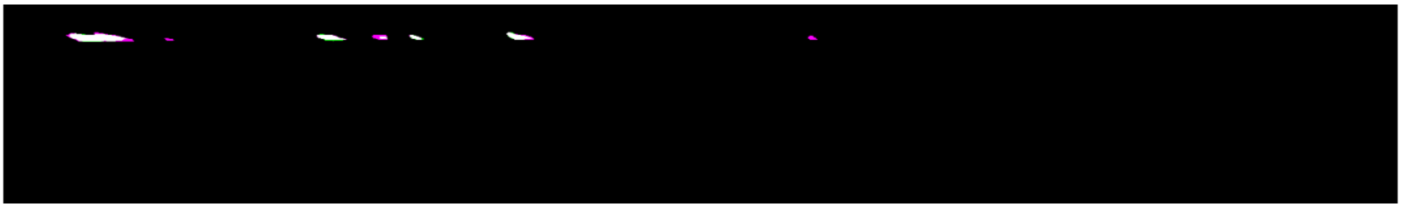

(b)

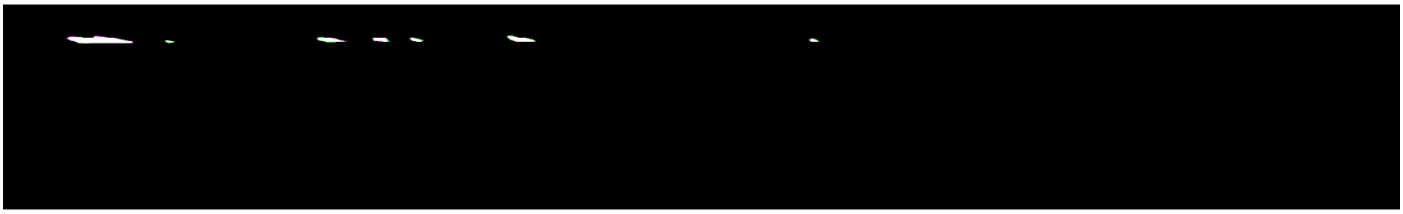

(c)

Figure S3. Examples of barb segmentation on moderate awn phenotype using original U-net and BarbNet: (a) original image, (b) segmentation using the original U-net vs. ground truth (DC=0.83), (c) segmentation using BarbNet vs ground truth (DC = 0.94). For the visualization purpose, original images were cropped to the region of interest.

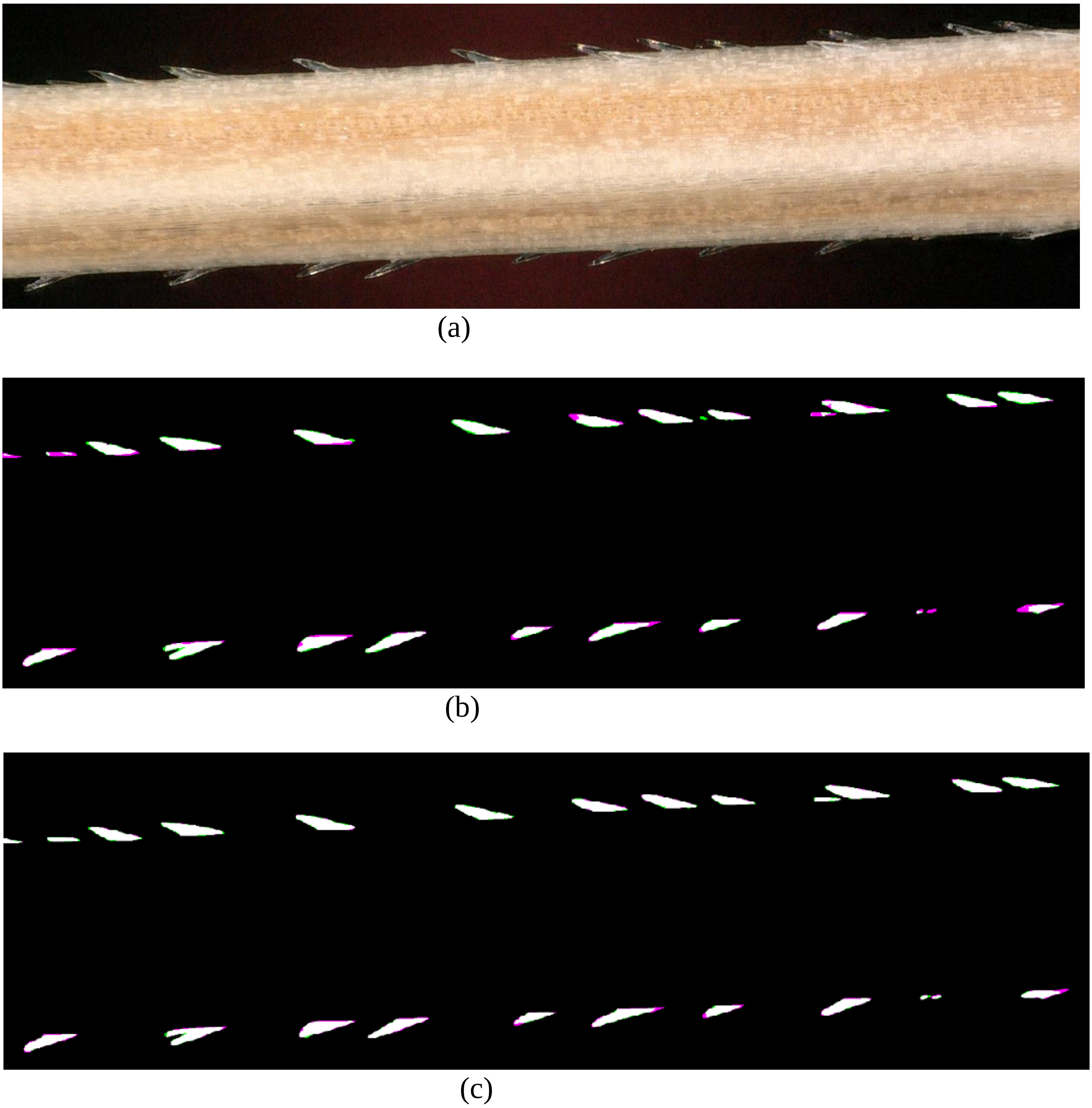

Figure S4. Examples of barb segmentation on dense awn phenotype using original U-net and BarbNet: (a) original image, (b) segmentation using the original U-net vs. ground truth (DC=0.88), (c) segmentation using BarbNet vs ground truth (DC = 0.95). For the visualization purpose, original images were cropped to the region of interest.

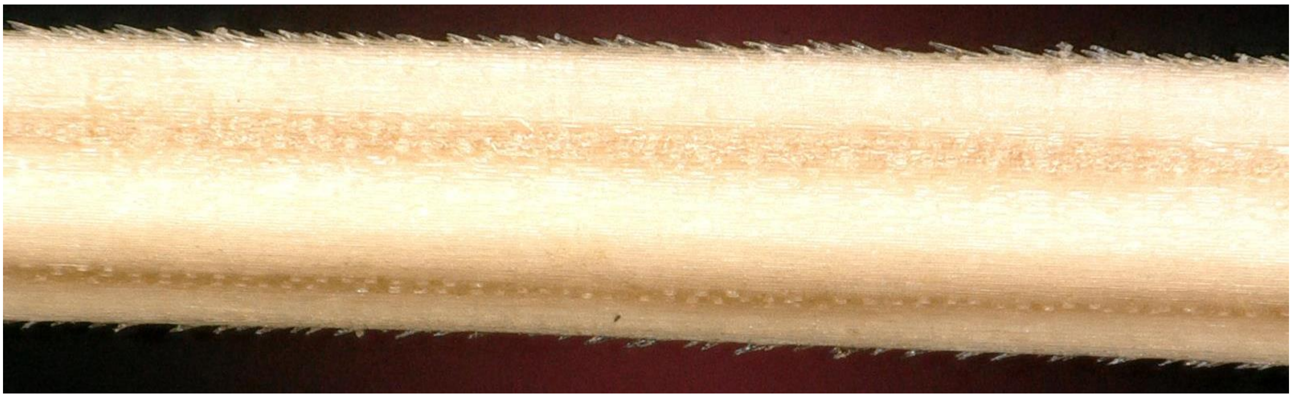

(a)

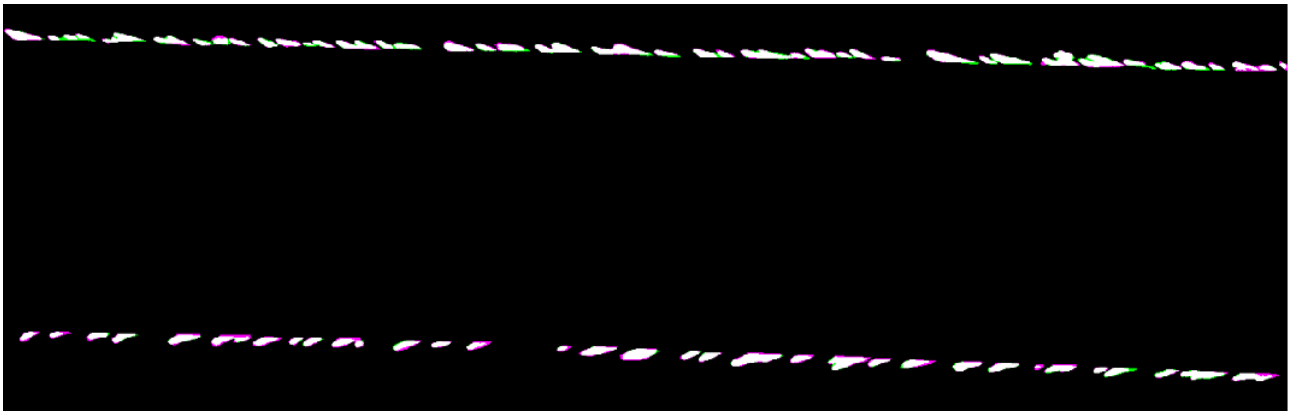

(b)

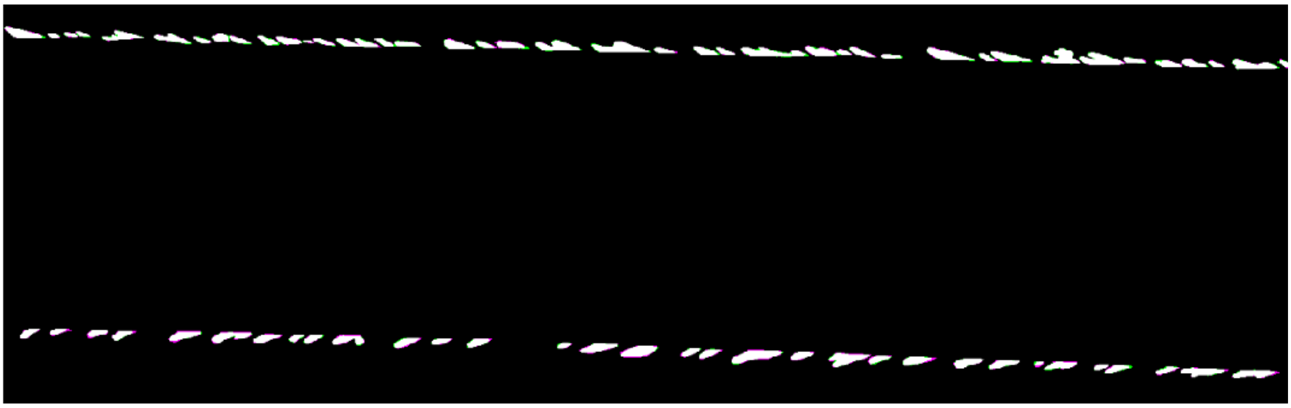

(c)
